# The Anti-Clot Treatment Scale (ACTS) in clinical trials: cross-cultural validation in venous thromboembolism patients

**DOI:** 10.1186/1477-7525-10-120

**Published:** 2012-09-26

**Authors:** Stefan J Cano, Donna L Lamping, Luke Bamber, Sarah Smith

**Affiliations:** 1 Department of Clinical Neuroscience, Peninsula College of Medicine and Dentistry, Room N16 ITTC Building, Tamar Science Park, Davy Road, Plymouth, Devon, PL6 8BX, UK; 2 Department of Health Services Research and Policy, London School of Hygiene and Tropical Medicine, Keppel Street, London, WC1E 7HT, UK; 3 Bayer Pharma AG, Wuppertal, Germany; 4 Department of Health Services Research and Policy, London School of Hygiene and Tropical Medicine, Keppel Street, London, WC1E 7HT, UK

**Keywords:** PRO instruments, Rating scales, Reliability, Validity, Venous thromboembolism

## Abstract

**Background:**

The Anti-Clot Treatment Scale (ACTS) is a 15-item patient-reported instrument of satisfaction with anticoagulant treatment. It includes a 12-item ACTS Burdens scale and a 3-item ACTS Benefits scale. Its role in clinical trials and other settings should be supported by evidence that it is both clinically meaningful and scientifically sound. The aim of the study was to evaluate the measurement performance of the ACTS (Dutch, Italian, French, German and English language versions) in patients with venous thromboembolism based on traditional psychometric methods.

**Methods:**

ACTS Burdens and Benefits scale data from a large clinical trial (EINSTEIN DVT) involving 1336 people with venous thromboembolism were analysed at both the scale and item level. Five key psychometric properties were examined using traditional psychometric methods: acceptability, scaling assumptions, reliability (including internal consistency reliability, test-retest reproducibility); validity (including known groups and discriminant validity); and responsiveness. These methods of examination underpin the US Food and Drug Administration recommendations for patient-reported outcome instrument evaluation.

**Results:**

Overall, the 12-item ACTS Burdens scale and 3-item ACTS Benefits scale met the psychometric criteria evaluated at both item and scale levels, with the exception of some relatively minor issues in the Dutch language version, which were just below reliability criteria (i.e. alpha = 0.72, test-retest intraclass correlation = 0.79). A consistent finding from item-level evaluations of aggregate endorsement frequencies and skewness suggested that response scales may be improved by reducing the number of response options from five to four.

**Conclusions:**

Both the ACTS Burdens and ACTS Benefits scales consistently satisfied traditional reliability and validity criteria across multiple language datasets, supporting it as a clinically useful patient-reported instrument of satisfaction with anticoagulant treatment in clinical trials.

**Trial registration number:**

NCT00440193

## Background

Patient-reported outcome (PRO) instruments are rapidly becoming the primary or secondary outcome measures of choice in pivotal clinical trials, research and practice [1], which means that PRO data now have a key role in patient care, policy-making and prescribing. The quality of inferences made from clinical trials is dependent on the PRO instruments used, and thus they need to be scientifically robust and clinically meaningful [2]. This is increasingly acknowledged [3,4] and has led the US Food and Drug Administration (FDA) to produce guidelines [5] that specify minimum criteria for the scientific adequacy of scales in clinical trials.

Venous thromboembolism (VTE) encompassing deep vein thrombosis (DVT) and pulmonary embolism (PE) occurs with an incidence rate of 1 to 2 per 1000 persons per annum in Western countries, with two-thirds of cases presenting with DVT [6]. VTE can be idiopathic in nature or be associated with risk factors such as surgery, limb trauma or cancer [7]. Oral anticoagulant therapy with vitamin K antagonists (VKAs), alongside initial parenteral heparin, have proved effective in the secondary prevention of recurrent VTE [8,9]. However, VKA treatment involves regular monitoring and dose adjustment, owing to a narrow therapeutic window and an inherent variability arising from genetic and dietary factors. This can be challenging for the patient with the potential to limit long-term persistence and adherence. In addition, bleeding is an important side-effect of anticoagulation. Therefore, as new anticoagulant therapies become available, it will be essential to measure not only their effectiveness and safety in improving clinical outcomes, but also their effectiveness in improving patient satisfaction [10,11].

The Anti-Clot Treatment Scale (ACTS) is a 15-item patient-reported instrument of satisfaction with anticoagulant treatment. It includes a 12-item ACTS Burdens scale and a 3-item ACTS Benefits scale. The ACTS also includes two additional global questions (see Appendix A). The ACTS was developed based on the original conceptual model of the Duke Anticoagulation Satisfaction Scale (DASS) following a literature review, interviews with experts and patients, and qualitative cognitive debriefing interviews [10-12]. The original DASS included 25 items covering the limitations, ‘hassles’ and positive impacts related to anticoagulant treatment. Modification of the DASS focused on making the instrument more applicable to a wider range of respondents – in particular, patients with DVT and PE and those in different country settings. This was achieved through qualitative research involving patient interviews and consensus panels (further information is available from the authors). The key changes included simplification of the wording and structure of the original instrument, improving item stems, changing the response timeframe, reducing the response categories from 7 to 5, and selecting the most relevant items for patients undergoing the different types of anticoagulant treatment. The focus of the new instrument is to delineate the burdens and benefits associated with anticoagulation therapy, and is designed to be used in patients receiving long-term anticoagulation irrespective of the underlying condition.

If the ACTS is to be considered suitable for future measurement of the burdens and benefits of anticoagulation therapy in patients with VTE, it should satisfy stringent criteria as a reliable and valid instrument. This study provides clinical researchers with a comprehensive evaluation of the reliability and validity of the ACTS using traditional psychometric methods in line with current guidelines.

## Methods

### Setting and participants

Bayer Pharma AG provided anonymised, blinded ACTS Burdens and Benefits scale datasets from EINSTEIN DVT, a large clinical trial involving patients with acute symptomatic DVT treated with rivaroxaban or enoxaparin/VKAs [13]. The inclusion criteria included: patients aged 18 years or older with a diagnosis of acute symptomatic DVT without symptomatic PE. The EINSTEIN DVT study included data from 1336 patients across six time points (day 15, 1 month, 2 months, 3 months, 6 months and 12 months). The protocols associated with the EINSTEIN trial programme were approved by the institutional review board at each centre and written informed consent was obtained from all patients. For the current psychometric analysis reported in this paper, the earliest time point data for each trial was analysed (i.e. the first time that patients completed the ACTS Burdens and Benefits scales; day 15).

Patients were asked to complete a questionnaire booklet containing the ACTS and Treatment Satisfaction Questionnaire for Medication version 2 (TSQM II) during follow-up visits. The measurement performance of the ACTS Burdens and Benefits scales was evaluated in the following languages: Dutch, Italian, French, German and English, and then a pooled dataset of all language versions. In this paper, the scale-level analyses for the separate study/language versions (acceptability, scaling assumptions and reliability, including internal consistency reliability, test-retest reproducibility) and both item and scale-level analysis for the pooled language versions datasets (acceptability, scaling assumptions, reliability [including internal consistency reliability]; validity [including known groups and discriminant validity]; and responsiveness) are presented. Further information is available from the authors.

### Instruments

The ACTS is a 15-item, patient-reported measure of satisfaction with anticoagulant treatment. It includes 12 items that assess the burdens of anticoagulant treatment and three items that assess the benefits of anticoagulant treatment. Patients are asked to rate their experiences of anticoagulant treatment during the past 4 weeks on a 5-point scale of intensity (1 = not at all, 2 = a little, 3 = moderately, 4 = quite a bit, 5 = extremely). The ACTS Burdens total score ranges from 12 to 60, and the ACTS Benefits total score ranges from 3 to 15. When used in clinical research, it is recommended that the ACTS Burdens scores are reverse-scored so that higher ACTS Burdens and Benefits scores indicate greater satisfaction with treatment. For the purposes of this psychometric evaluation, however, the original raw score data were analysed. French, Dutch, Italian, German and English language versions of the ACTS were created previously in accordance with a standard protocol to achieve conceptual equivalence in the translation, including: forward/backward translation, reconciliation, review and pilot testing [14]. Further information about the translation process is available from the authors.

For validation purposes, the TSQM II was also included. This is an 11-item PRO instrument that assesses patient satisfaction with treatment. It includes four scales: two items that assess the effectiveness of treatment (TSQM II Effectiveness), three items that assess side-effects (TSQM II Side-effects), three items that assess convenience of treatment (TSQM II Convenience) and two items that assess global satisfaction (TSQM II Global) [15]. Patients are asked to rate their experiences of treatment between ‘extremely dissatisfied’ and ‘extremely satisfied’ on 5-point to 7-point scales. Higher TSQM II scores indicate higher satisfaction with treatment.

### Data analysis

Psychometrics is a well-established scientific field that is concerned with the measurement of subjective judgements using numerical scales and the evaluation of the measurement properties of such scales (e.g. reliability, validity, responsiveness). The most widely used methods for evaluating measurement performance are known as ‘traditional’ psychometric methods [16]. Traditional psychometric methods form the basis for the recent FDA guidelines [1,2,5] that specify minimum criteria for the scientific adequacy of PRO instruments in clinical trials. The methods and criteria selected for evaluating the psychometric performance of the ACTS are grounded in current widely accepted guidelines [3,4,17-20], including the FDA guidance [5]. This methodology has been used extensively in previous research to develop and validate PRO instruments in other areas of medicine and surgery [21-24].

Based on data collected, the following psychometric properties of the ACTS were examined: acceptability (including data quality and targeting); scaling assumptions; internal consistency reliability and test-retest reproducibility; aspects of validity (including known groups and discriminant validity); and responsiveness. Table 1 summarises the psychometric methods and criteria used in this study to analyse and interpret results. Acceptability and reliability analyses were carried out on the separate language versions of the ACTS and the combined sample at baseline (N = 1336). Factor analysis and item convergent/discriminant validity analysis were conducted on the combined sample at baseline (N = 1336). Test-retest reproducibility and construct validity examinations comparing the TSQM II were conducted on ACTS data from a separate sub-sample of patients at 3 months (Burdens scale, n = 792; Benefits scale, n = 822). Responsiveness analysis was carried out on ACTS data from a separate sub-sample of patients who completed the ACTS at baseline and 3 months (Burdens scale, n = 1227; Benefits scale, n = 1257).

**Table 1:** Summary of psychometric methods

**Psychometric property**	**Definition**/**criteria for acceptability**
Acceptability	Assessed by data quality and targeting. Data quality refers to the completeness of item- and scale-level data. Assessed by completeness of data; criterion for missing data <10% [20]. Targeting is the extent to which the range of the variable measured by a scale matches the range of that variable in the study sample. Assessed by: maximum endorsement frequencies <80% [17], aggregate endorsement frequencies^a^ >10% [17], and skewness statistic −1 to +1 [35-37], proximity of scale mean score to scale midpoint^b^ (no fixed criterion but closer matches indicated better targeting) [38], and acceptable distribution of ACTS Burdens scores^c^ (no fixed criterion but closer to 100% indicates better targeting) [39]
Scaling assumptions	Tests of scaling assumptions assess the extent to which it is legitimate to sum a set of items, without weighting or standardisation, to produce a single total score. This criterion is satisfied when items have adequate corrected-item total correlations ≥0.30 [38,40] and the proposed grouping of items in each subscale is correct. Assessed by using two complementary approaches: principal components analysis (factor loadings >0.30, cross-loadings <0.20) and item convergent and discriminant validity (item own-scale correlations >0.30, magnitude >2 standard errors than other scales)
Reliability	Reliability is the extent to which scale scores are not associated with random error
*Internal consistency reliability*	The precision of the scale based on the homogeneity (intercorrelations) of items at a single point in time. Assessed using Cronbach’s alpha ≥0.80 [41,42], mean item-item correlations (known as the homogeneity coefficient) ≥0.30 [37] and item-total correlations ≥0.30 [42]
*Test*-*retest reproducibility*	This is based on the agreement between people scores at screening and baseline, and estimates the ability of components and scales to produce stable scores [34]. For adequate test-retest reproducibility, scale-level intraclass correlation coefficients ≥0.80 [40] and item-level intraclass correlation coefficients ≥0.50 [43] should be achieved
Validity	Validity is the extent to which a scale measures the construct that it is intended to measure
*Validity* (*within scale)*	Evidence that a scale measures a single construct, and that items can be combined to form a summary score. Assessed on the basis of internal consistency reliability (Cronbach’s alpha ≥ 0.80) and factor analysis (factor loadings >0.30, cross-loadings < 0.20)
*Validity (correlations between scales)*	Correlations between ACTS scales: moderate correlations (0.30–0.70) expected. Correlations between TSQM II^d^[44] and ACTS scales: low correlations (< 0.30) expected between TSQM II Effectiveness and ACTS Burdens/ACTS Benefits; low correlations (< 0.30) expected between TSQM II Side-effects and ACTS Benefits; moderate correlations (0.30–0.70) expected between TSQM II Side-effects and ACTS Burdens; moderate correlations (0.30–0.70) expected between TSQM II Convenience and ACTS Burdens/ACTS Benefits; moderate correlations (0.30–0.70) expected between TSQM II Global Satisfaction and ACTS Burdens/ACTS Benefits
*Discriminant validity*	Evidence that a scale is not correlated with other measures of different constructs. Assessed on the basis of correlations between the ACTS and age and gender; low correlations (<0.30) expected between ACTS scores and age and gender
*Known-groups validity/hypothesis testing*	Ability of a scale to detect hypothesised differences between known subgroups. Assessed by testing the hypothesis that known groups defined on the basis of high vs low ACTS global scores for: i) Burdens (Q13) and ii) Benefits (Q17) will differ significantly (in the expected direction) on ACTS Burdens and Benefits scale scores; based on ANOVA (p<0.05)
Responsiveness	The ability of the ACTS Burden and Benefits scales to detect significant change over time, assessed by examining scores at two or more time points of surgery and calculating an effect size statistic calculated as the mean difference (change score) in scores at time point 1 to time point 2 divided by the standard deviation of the time 1 score [44]. Clinically, increasing moderate effect sizes over time would be expected, reflecting improved treatment satisfaction. Effect sizes were interpreted as the following: 0.20 (small change), 0.50 (moderate change) and >0.80 (large change) [45]

^a^Calculated as the sum of responses between any two adjacent response categories (e.g. if responses to ‘not at all’ = 2% and ‘a little’ = 7%, aggregate endorsement frequency = 9%, which fails the criterion).

^b^Calculated as possible scale midpoint minus actual scale mean score.

^c^Calculated as actual scale range divided by possible scale range multiplied by 100.

^d^TSQM II was designed as a general measure of treatment satisfaction with medication, and includes 11 items in 4 sub-scales (Effectiveness, Side-effects, Convenience, Global Satisfaction).

*ACTS* Anti-Clot Treatment Scale, *ANOVA* analysis of variance, *TSQM II* Treatment Satisfaction Questionnaire for Medication version 2.

## Results

### Sample

The EINSTEIN DVT ACTS validation dataset included 1336 patients (96% response rate) at day 15 (average age was 57 years [standard deviation (SD) 16] and 42% were female). Across each of the questionnaire language versions, this comprised: Dutch (n = 332; 55 years [SD = 15], 41% female); Italian (n = 217; 64 years [SD = 16], 41% female); French (n = 222; 57 years [SD = 18], 41% female); German (n = 243; 56 years [SD = 15], 40% female); and English (UK, US, Canada; n = 322; 54 years [SD = 15], 44% female). The pooled EINSTEIN DVT dataset, including all language versions, comprised 1336 patients (average age was 57 years [SD = 16] and 42% were female). Overall, patients participating in the treatment satisfaction sub-study had similar baseline characteristics to the full EINSTEIN DVT trial population (a full description of patient demographics is presented elsewhere).

### Psychometric properties: scale level by study/language version (Dutch, Italian, French, German, English)

#### Acceptability: data quality and targeting

For each language version, there was a low level of missing data for all item and scale scores for both the ACTS Burdens and ACTS Benefits (scale level <5%). This means that scale scores could be computed for >95% of patients. There was a reasonable distribution of ACTS Burdens scores (mean 60%, range 54–77%), and an excellent distribution of ACTS Benefits scores (mean 100%). Floor and ceiling effects were generally low for both scale scores (mean 5%, range 0–14%) and data skewness was slightly higher for the ACTS Burdens scores than for the ACTS Benefits scores (mean −1.02 [range −0.78 to −1.35] vs −0.84 [range −1.97 to 0.03], respectively) (Table 2).

**Table 2:** ACTS Burdens and Benefits scales: scale-level data quality, scaling assumptions, targeting and reliability

	**Acceptability**						**Targeting**		**Reliability**		
	**Item missing data** (**%)**^**a**^	**Possible range (midpoint)**	**Actual score range**	**Mean score (SD)**	**Floor/ceiling effects (%)**^**b**^	**Skewness**	**Cronbach’s alpha**	**Test-retest**^**c**^	**Mean IIC**^**d**^	**Range IIC**
**EINSTEIN DVT dataset**										
**Dutch version (n=332)**										
*ACTS Burdens*	4	12–60 (36)	34–60	53.4 (5.45)	0/10	–0.99	0.79	0.95	0.24	0.03–0.69
*ACTS Benefits*	1	3–15 (9)	3–15	10.5 (2.20)	1/3	−0.77	0.80	0.72	0.56	0.46–0.66
**Italian version (n=217)**										
*ACTS Burdens*	4	12–60 (36)	33–60	52.4 (6.26)	0/12	−0.78	0.90	0.94	0.43	0.25–0.83
*ACTS Benefits*	1	3–15 (9)	3–15	10.7 (2.15)	1/8	0.03	0.90	0.94	0.75	0.68–0.79
**French version (n=222)**										
*ACTS Burdens*	4	12–60 (36)	33–60	55.6 (4.97)	0/14	−1.35	0.89	0.95	0.40	0.23–0.70
*ACTS Benefits*	3	3–15 (9)	3–15	11.5 (2.40)	2/12	−0.89	0.86	0.76	0.68	0.60–0.72
**German version (n=243)**										
*ACTS Burdens*	5	12–60 (36)	34–60	52.0 (5.91)	0/7	−0.86	0.84	0.98	0.29	0.04–0.86
*ACTS Benefits*	1	3–15 (9)	3–15	12.2 (2.15)	2/12	−1.97	0.82	0.91	0.60	0.56–0.67
**English version (n=322)**^**e**^										
*ACTS Burdens*	0	12–60 (36)	23–60	51.6 (6.78)	0/7	−1.12	0.86	0.98	0.36	0.12–0.67
*ACTS Benefits*	0	3–15 (9)	3–15	11.4 (2.50)	1/14	−0.62	0.87	0.91	0.70	0.59–0.74

^a^Less than 0.5% missing data rounded to 0. ^b^Calculated as the percentage of people scoring either 12 (floor) or 60 (ceiling) on the *ACTS Burdens* scale or 3 (floor) or 15 (ceiling) on the *ACTS Benefits* scale. ^c^Test-retest between two administrations at 12 weeks. ^d^Representing homogeneity. ^e^UK, US, Canada.

*ACTS* Anti-Clot Treatment Scale, *IIC* item-item correlation, *SD* standard deviation, *VTE* venous thromboembolism.

#### Reliability: internal consistency, test-retest and homogeneity coefficients

Across EINSTEIN DVT datasets, Cronbach’s alpha and test-retest intra-class correlations for both ACTS Burdens and ACTS Benefits scores were acceptable (>0.82), with the exception of the Dutch version (alpha = 0.79; test-retest = 0.72). The homogeneity coefficient mean ranged from 0.24 to 0.75 (Table 2).

### Psychometric properties: item and scale level by combined language versions (EINSTEIN DVT pooled language datasets)

#### Acceptability: data quality and targeting

There were minimal missing data for all item and scale scores (<4%). Therefore, scale scores could be computed for >96% of patients, which was slightly higher than the individual country analysis owing to the effect of pooling the datasets. At the scale level, there was a good distribution of ACTS Burdens scores (77%) and an excellent distribution of ACTS Benefits scores (100%). Scale-level floor and ceiling effects were generally low for both scale scores (range 0–11%). Data skewness was slightly higher for the ACTS Burdens scale scores than for the ACTS Benefits scale scores (sk = −1.08 and sk = −0.80, respectively). At the item level, the ACTS Burdens scale ceiling effects ranged from 37% to 77%. In both datasets, in relation to aggregate endorsement frequencies, for all items, three of five response categories met the >10% criterion, but two of five response categories were <10% (between response categories 4 and 5) and 10 of 12 items fell outside the skewness criterion (−1, +1). The ACTS Benefits scale had much lower ceiling effects, which ranged from 15% to 19%. In relation to aggregate endorsement frequencies, for all items, three of five response categories met the >10% criterion, but two of five response categories were <10% (between response categories 4 and 5) and all items passed the skewness criterion (Table 3).

**Table 3:** ACTS Burdens and Benefits scales - EINSTEIN DVT dataset (all countries): data quality, scaling assumptions, targeting, reliability (N=1336)

	**Data quality**	**Scaling assumptions**					**Targeting**		**Reliability**			
	**Item missing data (%)**^**a**^	**Possible range (midpoint)**	**Actual score range**	**Mean score**	**SD**	**CITC**	**Floor/ceiling effects (%)**^**b**^	**Skewness**	**Cronbach’s alpha**	**Test-retest**^**c**^	**Mean IIC**^**d**^	**Range IIC**
**Burden items**												
Q1 Bleeding/vigorous activities	1	1–5	1–5	4.26	1.03	0.50	3/57	−1.41	-	-	-	-
Q2 Bleeding/usual activities	1	1–5	1–5	4.47	0.86	0.50	1/65	−1.79	-	-	-	-
Q3 Bruising	0	1–5	1–5	4.39	0.87	0.45	1/58	−1.49	-	-	-	-
Q4 Avoid other medicines	0	1–5	1–5	4.54	0.80	0.39	1/69	−1.84	-	-	-	-
Q5 Limit eat/drink	0	1–5	1–5	4.55	0.78	0.46	1/69	−1.87	-	-	-	-
Q6 Hassle/daily	0	1–5	1–5	4.41	0.78	0.60	0/56	−1.22	-	-	-	-
Q7 Hassle/occasional	1	1–5	1–5	4.02	0.98	0.57	2/38	−0.85	-	-	-	-
Q8 Difficult to follow your ACT	0	1–5	1–5	4.71	0.58	0.52	0/77	−2.19	-	-	-	-
Q9 Time-consuming ACT	0	1–5	1–5	4.52	0.69	0.43	0/62	−1.40	-	-	-	-
Q10 Worry about ACT	1	1–5	1–5	4.07	0.91	0.56	1/37	−0.84	-	-	-	-
Q11 Frustrating ACT	0	1–5	1–5	4.45	0.87	0.61	1/64	−1.76	-	-	-	-
Q12 Burden of ACT	1	1–5	1–5	4.49	0.75	0.65	0/62	−1.52	-	-	-	-
**ACTS Burdens scale**	4	12–60 (36)	23–60	52.9	6.1	-	0/11	−1.08	0.85	0.97	0.32	0.13–0.66
**Benefit items**												
Q14 Confident in ACT	1	1–5	1–5	3.77	0.91	0.71	3/19	−0.88	-	-	-	-
Q15 Reassured by ACT	1	1–5	1–5	3.71	0.87	0.80	3/15	−0.84	-	-	-	-
Q16 Satisfied with ACT	0	1–5	1–5	3.79	0.90	0.69	3/19	−0.92	-	-	-	-
**ACTS Benefits scale**	1	3–15 (9)	3–15	11.3	2.4	-	1/10	−0.80	0.86	0.86	0.67	0.59–0.73

^a^Less than 0.5% missing data rounded to 0. ^b^Calculated as the percentage of people scoring either 12 (floor) or 60 (ceiling) on the *ACTS Burdens* scale or 3 (floor) or 15 (ceiling) on the *ACTS Benefits* scale. ^c^Test-retest between two administrations at 12 weeks. ^d^Representing homogeneity.

*ACT* anticoagulation treatment, *ACTS* Anti-Clot Treatment Scale, *CITC* corrected item-total correlation, *IIC* item-item correlation, *SD* standard deviation, - analyses not conducted owing to lack of data.

### Psychometric properties: scaling assumptions

Item groupings in the ACTS Burdens and ACTS Benefits scales passed tests for scaling assumptions. Corrected-item total correlations for both scales ranged from 0.39 to 0.80, satisfying the recommended criteria (>0.30). This indicated that items in each scale measured a common underlying construct and contained a similar proportion of information. In addition, principal components analysis factor loadings (>0.48) and tests of item convergent/discriminant validity (>0.39) supported this finding, thus further indicating that all items in each of the scales passed the criteria (Tables 3, 4 and 5).

**Table 4:** ACTS Burdens and Benefits scales – EINSTEIN DVT datasets: principal components analysis with VARIMAX rotation

**Item**	**EINSTEIN DVT (N=1336)**	
	**Component**	
	**1**	**2**
**ACTS Burdens**		
Q1 Bleeding/vigorous activities	0.58	−0.06
Q2 Bleeding/usual activities	0.57	0.03
Q3 Bruising	0.54	0.06
Q4 Avoid other medicines	0.48	0.07
Q5 Limit eat/drink	0.55	0.02
Q6 Hassle/daily	0.69	0.10
Q7 Hassle/occasional	0.66	0.08
Q8 Difficult to follow your ACT	0.62	0.11
Q9 Time-consuming ACT	0.53	0.12
Q10 Worry about ACT	0.65	0.11
Q11 Frustrating ACT	0.71	0.09
Q12 Burden of ACT	0.74	0.09
**ACTS Benefits**		
Q14 Confident in ACT	0.03	0.88
Q15 Reassured by ACT	0.07	0.91
Q16 Satisfied with ACT	0.19	0.83

Extraction method: principal components analysis; rotation method: VARIMAX with Kaiser normalization.

*ACT* anticoagulation treatment, *ACTS* Anti-Clot Treatment Scale.

Values refer to factor loadings on each component: Component 1 = ACTS Burdens; Component 2 = ACTS Benefits.

**Table 5:** ACTS Burdens and Benefits scales – EINSTEIN DVT dataset: item convergent/discriminant validity

**Item**	**EINSTEIN DVT (N=1336)**	
	**ACTS Burdens scale**	**ACTS Benefits scale**
**ACTS Burdens**		
Q1 Bleeding/vigorous activities	0.50	0.06
Q2 Bleeding/usual activities	0.50	0.14
Q3 Bruising	0.45	0.13
Q4 Avoid other medicines	0.39	0.12
Q5 Limit eat/drink	0.46	0.10
Q6 Hassle/daily	0.60	0.17
Q7 Hassle/occasional	0.57	0.15
Q8 Difficult to follow your ACT	0.52	0.16
Q9 Time-consuming ACT	0.43	0.15
Q10 Worry about ACT	0.56	0.18
Q11 Frustrating ACT	0.61	0.17
Q12 Burden of ACT	0.65	0.16
**ACTS Benefits**		
Q14 Confident in ACT	0.13	0.71
Q15 Reassured by ACT	0.17	0.80
Q16 Satisfied with ACT	0.26	0.69

*ACT* anticoagulation treatment, *ACTS* Anti-Clot Treatment Scale.

Values refer to correlations of items to their own scale (corrected-item total correlations) and other scales (Pearson’s r correlations).

### Psychometric properties: internal consistency reliability

Corrected-item total correlations (>0.39), Cronbach’s alpha (>0.85) and test-retest intraclass correlations (>0.86) for both scales in both datasets passed the criteria, supporting their reliability (Table 3).

### Psychometric properties: validity

Overall, the correlations with the four TSQM II scale scores were consistent with predictions (4/4 correlations meeting predictions; Table 6). Known groups validity was supported for both the ACTS Burdens and ACTS Benefits scale scores on the global items (p < 0.0001; further information available from the authors). Discriminant validity correlations suggest no bias by age or sex (r < −0.16).

**Table 6:** ACTS Burdens and Benefits scales – EINSTEIN DVT dataset: construct validity correlations with TSQM II subscales at 3 months

	**ACTS Burdens**	**ACTS Benefits**	**TSQEFF**	**TSQSIDE**	**TSQCON**
ACTS Burdens	1.00				
ACTS Benefits	0.33	1.00			
TSQEFF	0.16	0.18	1.00		
TSQSIDE	0.35	0.14	0.29	1.00	
TSQCON	0.32	0.24	0.53	0.29	1.00
TSQGLO	0.32	0.27	0.53	0.36	0.74

*ACTS* Anti-Clot Treatment Scale, *TSQCON* TSQM II Convenience, *TSQEFF* TSQM II Effectiveness, *TSQGLO* TSQM II Global Satisfaction, *TSQSIDE*, TSQM II Side-effects.

### Psychometric properties: responsiveness

The pattern of mean scores over time suggested a trend to higher scores in the ACTS Burdens and ACTS Benefits scales over the six time points assessed (day 15, 1 month, 2 months, 3 months, 6 months, and 12 months). Responsiveness statistics comparing day 15 scores with all the other time points individually supported a trend of increasingly higher ACTS Burdens and Benefits scales scores over time, with low but increasing effect size statistics (range −0.14 to −0.37 and −0.03 to −0.33, respectively) (Table 7).

**Table 7:** ACTS Burdens and Benefits scales – EINSTEIN DVT dataset (N=1336): responsiveness – mean change score, t statistic, p-value and effect size for day 15 compared with all other time points

		**Mean**	**SD**	**t**	**P**	**ES**
Pair 1	ACTS Burdens day 15 to ACTS Burdens month 1	−0.84	3.98	7.30	0.000	−0.14
Pair 2	ACTS Burdens day 15 to ACTS Burdens month 2	−1.25	4.75	8.95	0.000	−0.20
Pair 3	ACTS Burdens day 15 to ACTS Burdens month 3	−1.35	4.65	9.80	0.000	−0.22
Pair 4	ACTS Burdens day 15 to ACTS Burdens month 6	−1.65	4.88	10.39	0.000	−0.27
Pair 5	ACTS Burdens day 15 to ACTS Burdens month 12	−2.00	4.40	5.78	0.000	−0.37
Pair 1	ACTS Benefits day 15 to ACTS Benefits month 1	−0.06	2.22	−1.03	0.304	−0.03
Pair 2	ACTS Benefits day 15 to ACTS Benefits month 2	−0.16	2.45	−2.30	0.021	−0.07
Pair 3	ACTS Benefits day 15 to ACTS Benefits month 3	−0.18	2.46	−2.58	0.010	−0.08
Pair 4	ACTS Benefits day 15 to ACTS Benefits month 6	−0.29	2.49	−3.71	0.000	−0.12
Pair 5	ACTS Benefits day 15 to ACTS Benefits month 12	−0.77	2.60	−3.84	0.000	−0.33

*ACTS* Anti-Clot Treatment Scale, *SD* standard deviation.

## Discussion

Current PRO instrument guidelines [3-5] make it increasingly important for clinical researchers to understand the science behind the instruments used to try to capture the patient perspective. In this study, both the ACTS Burdens and ACTS Benefits scales satisfied traditional psychometric criteria for data quality, scaling assumptions, targeting, reliability, validity and responsiveness. In fact, its psychometric properties were found to be remarkably stable across different cultural groups, supporting pooling of data. This study, together with previous work on conceptual model development [10,11], provides an initial evidence base for its use in clinical trials and other settings (e.g. post-market surveillance, clinical research and in practice), in line with the current FDA guidelines (Table 8). The ACTS can be used to evaluate and compare different therapies in patients with DVT [25], it is acceptable to patients, and has a simple checklist format that can be completed easily and quickly. Importantly, the ACTS measures aspects of treatment satisfaction, treatment adherence, relevance (e.g. burdens surrounding treatment regimens, impact on daily activities, and the possibility of bruising and bleeding) and important positive outcomes to patients (e.g. benefits surrounding assurance and confidence in treatment) [11].

**Table 8:** Adapted from Table 4 of the FDA guidelines for measurement properties reviewed for PRO instruments used in clinical trials

**Measurement property**	**Test**	**Methods used in testing the ACTS**
Reliability	Test-retest	✓
Internal consistency	Whether the items in a domain are intercorrelated, as evidenced by an internal consistency statistic (e.g., coefficient alpha)	✓
Inter-interviewer reproducibility (for interviewer-administered PROs only)	Agreement between responses when the PRO is administered by two or more different interviewers	NA
Validity	Content-related	✓^a^
Ability to measure the concept (also known as construct-related validity; can include tests for discriminant, convergent and known-groups validity)	Whether relationships among items, domains and concepts conform to what is predicted by the conceptual framework for the PRO instrument itself and its validation hypotheses	✓
Ability to predict future outcomes (also known as predictive validity)	Whether future events or status can be predicted by changes in the PRO scores	x
Ability to detect change	Includes calculations of effect size and standard error of measurement among others	✓
Interpretability	Smallest difference that is considered to be clinically important; this can be a specified difference (the minimum important difference) or, in some cases, any detectable difference. The minimum important difference is used as a benchmark to interpret mean score differences between treatment arms in a clinical trial	✓^b^
Responder definition – used to identify responders in clinical trials for analysing differences in the proportion of responders between treatment arms	Change in score that would be clear evidence that an individual patient experienced a treatment benefit. Can be based on experience with the measure using a distribution-based approach, a clinical or non-clinical anchor, an empirical rule, or a combination of approaches	NA

^a^Reported in Wild *et al*. 2009 [10]; ^b^Further additional information available from the authors.

✓=tested; x=not tested.

*ACTS* Anti-Clot Treatment Scale, *FDA* US Food and Drug Administration, *NA* not applicable, *PRO* patient-reported outcome.

Overall, the 12-item ACTS Burdens and 3-item ACTS Benefits scales met the psychometric criteria evaluated at both item and scale levels. Item-level targeting was adequate given the nature of the target construct (i.e. a scale that taps into aspects of treatment satisfaction would be expected to result in a degree of skew to the positive in score distributions) [26]. Scaling assumptions were also broadly supported, as were criteria for internal consistency reliability at item and scale level and scale-level test-retest reproducibility. Validity was also supported by assessments of discriminant validity and known-groups comparisons. Finally, responsiveness analyses supported increasing improvement over time in both treatment satisfaction scores. Looking forward, three areas require further consideration: construct validity, response options and further exploration of responsiveness.

First, construct validity analyses in the form of testing hypothesised correlations between the ACTS Burdens scale and four scales of the TSQM II were supported in the pooled dataset but were slightly lower than expected. This issue may reflect the fact that, although both measures focus on treatment satisfaction, the constructs captured by the ACTS and TSQM II are more distinct than would be first expected. On closer inspection, the TSQM II items that capture ‘Effectiveness’, ‘Side-effects’, ‘Convenience’ and ‘Global satisfaction’ are significantly different from the ACTS Burdens and Benefits items. Thus, despite some overlap between the two instruments, there are key differences – for example, the TSQM II Effectiveness scale has one of two items addressing symptom alleviation, not directly impacted by anti-coagulation or measured by ACTS. Furthermore, the TSQM II Side-effects items do not directly address the important anticoagulation-specific side-effects of bleeding or bruising, which are measured in ACTS Burdens items. In addition, the TSQM II is more narrowly targeted at the medicine, the ACTS being more inclusive of the services and difficulties of undergoing anticoagulation therapy. Thus, the findings from the analyses should be interpreted with these facts in mind.

The second issue that requires further exploration is that, across all language versions, findings from item-level tests of aggregate endorsement frequencies and skewness suggested that response scales may be improved by reducing the number of response options from five to four. This is also reflected in the findings from the scale-level targeting, which also revealed slightly skewed distributions across the board. One potential cause for this is that there may simply have been too many response options for respondents to discriminate between, especially at the more satisfied extreme of the choices. This is not uncommon in PRO instruments [27] and it has been found previously that four categories work better than five [28]. Another possibility is that the response category labelling is problematic. The present findings uncovered a consistent issue in the way in which patients responded in the ‘not at all’ and ‘a little’ categories. Therefore, a reconsideration of wording in these response categories may also help to improve measurement performance. However, given that the current validation is limited to the five-option response scale, this is a matter for consideration in future development of the ACTS.

The responsiveness analyses, which can be considered to be preliminary, revealed a modest but stepwise increase in ACTS Burdens and Benefits scale scores over the six time points. The associated responsiveness statistics were moderate but were in the range that would be expected clinically. This is because scale responsiveness and treatment effectiveness are inseparably linked [29,30]. The effect sizes computed on ACTS Burdens and Benefits scale scores from day 15 to all other time points are indicators of the ability of these scales to detect change. However, these are also an indicator of the size of the treatment effect. To put the present findings in context, it may be useful to consider the effect sizes of other interventions. Thus, effect sizes associated with hip arthroplasty have been shown to be very large (3.1) [31]. This would be expected given the dramatic impact of this surgical intervention of pain symptomatology. By contrast, the effect of carpal tunnel repair on grip strength is small (0.2) [32]. A degree of improved treatment satisfaction associated with anticoagulant treatment would be expected to occur over time, but would not be expected to be as marked as intensive interventions. However, the clinical meaning of the ACTS Burdens and Benefits scale change scores and specification of what constitutes an important difference based on these scores are matters for consideration in future development of the ACTS. Given some of the potential limitations of traditional responsiveness statistics [33], further evaluations would be desirable using more sophisticated modern rating scale analysis techniques [34] to further delineate the specific ability of the ACTS Burdens and Benefits to detect differences between and clinically meaningful change within patients.

Our study has two key limitations. First, although the scope of our psychometric evaluation of the ACTS in patients being treated for acute DVT was relatively comprehensive, there are further analyses that would aid our understanding of the measurement performance of the ACTS Burdens and ACTS Benefits scales. These would include further known groups and discriminant validity tests based on clinically sensible sub-grouping (based on predefined hypothesis-driven selection) and responsiveness analyses assessed against *a priori* clinically anchored hypotheses. The second limitation is that the small size of the English-language versions (i.e. US, UK, Canada) required that these be combined prior to psychometric analyses. Further testing using adequate samples in each country would provide useful additional evidence for the relative measurement performance of the ACTS scales.

## Conclusions

Overall, across the different language versions, evidence from the psychometric evaluation of the ACTS in patients treated for acute DVT supports the use of the 12-item ACTS Burdens scale and the 3-item ACTS Benefits scale. The ACTS can provide essential information about VTE-related treatment satisfaction from the patient’s perspective. This new instrument will complement current clinical outcome measures and facilitate multicentre studies for comparison of treatments and patient populations. Thus, the ACTS has the potential to support treatment trials, cost-effectiveness analysis and patient education, providing crucial data for clinical researchers, clinicians and patients. Further potential psychometric research on the ACTS includes exploring reducing the number of response options from five to four, providing evidence that both ACTS Burdens and Benefits scales are able to detect a clinically meaningful change over time and exploring the potential of using the scales in clinical practice settings.

## Appendix A. Anti-Clot Treatment Scale

During the past 4 weeks…

1 How much does the possibility of bleeding as a result of anti-clot treatment limit you from taking part in vigorous physical activities? (e.g. exercise, sports, dancing, etc.).

2 How much does the possibility of bleeding as a result of anti-clot treatment limit you from taking part in your usual activities? (e.g. work, shopping, housework, etc.).

3 How bothered are you by the possibility of bruising as a result of anti-clot treatment?

4 How bothered are you by having to avoid other medicines (e.g. aspirin) as a result of anti-clot treatment?

5 How much does anti-clot treatment limit your diet? (e.g. food or drink, including alcohol).

6 How much of a hassle (inconvenience) are the daily aspects of anti-clot treatment? (e.g. remembering to take your medicine at a certain time, taking the correct dose of your medicine, following a diet, limiting alcohol, etc.).

7 How much of a hassle (inconvenience) are the occasional aspects of anti-clot treatment? (e.g. the need for blood tests, going to or contacting the clinic/doctor, making arrangements for treatment while travelling, etc.).

Now I want to ask you about daily and occasional aspects of your anticoagulation therapy during the past 4 weeks

8 How difficult is it to follow your anti-clot treatment?

9 How time-consuming is your anti-clot treatment?

10 How much do you worry about your anti-clot treatment?

11 How frustrating is your anti-clot treatment?

12 How much of a burden is your anti-clot treatment?

13 Overall, how much of a negative impact has your anti-clot treatment had on your life?

14 How confident are you that your anti-clot treatment will protect your health? (e.g. prevent blood clots, stroke, heart attack, DVT, embolism)

15 How reassured do you feel because of your anti-clot treatment?

16 How satisfied are you with your anti-clot treatment?

17 Overall, how much of a positive impact has your anti-clot treatment had on your life?

## Abbreviations

ACTS: Anti-Clot Treatment Scale; DASS: Duke Anticoagulation Satisfaction Scale; DVT: Deep vein thrombosis; FDA: US Food and Drug Administration; PE: Pulmonary embolism; PRO: Patient-reported outcome; SD: Standard deviation; TSQM II: Treatment Satisfaction Questionnaire for Medication version 2; VKA: Vitamin K antagonist; VTE: Venous thromboembolism.

## Competing interests

LB is an employee of Bayer Pharma AG. SC, DL and SS were supported in part through a grant from Bayer Pharma AG.

## Authors’ contributions

SC conducted, analysed and interpreted the data and wrote the manuscript. DL was involved in guiding the study, including design and analysis of data. LB and SS were involved in providing additional input and reviewing drafts of this manuscript. All authors read and approved the final manuscript.

## Copyright

Copyright of the ACTS instrument is held by Bayer AG, Germany (2006). All rights reserved. For information on or permission to use, please contact Mapi Research Trust; http://www.mapi-trust.org.
